# Overview of *Proteus mirabilis* pathogenicity and virulence. Insights into the role of metals

**DOI:** 10.3389/fmicb.2024.1383618

**Published:** 2024-04-05

**Authors:** Mohamed Chakkour, Zeinab Hammoud, Solay Farhat, Ali El Roz, Zeinab Ezzeddine, Ghassan Ghssein

**Affiliations:** ^1^Department of Biological Sciences, Wayne State University, Detroit, MI, United States; ^2^Faculty of Medical Sciences, Lebanese University, Beirut, Lebanon; ^3^Laboratory Sciences Department, Faculty of Public Health, Islamic University of Lebanon (IUL), Khalde, Lebanon

**Keywords:** *Proteus mirabilis*, epidemiology, virulence factors, siderophores, bacteria

## Abstract

*Proteus mirabilis* is a Gram-negative bacterium with exclusive molecular and biological features. It is a versatile pathogen acclaimed for its distinct urease production, swarming behavior, and rapid multicellular activity. Clinically, *P. mirabilis* is a frequent pathogen of the human urinary system where it causes urinary tract infections (UTIs) and catheter-associated urinary tract infections (CAUTIs). This review explores the epidemiology, risk factors, clinical manifestations, and treatment of *P. mirabilis* infections, emphasizing its association with UTIs. The bacterium’s genome analysis revealed the presence of resistance genes against commonly used antibiotics, an antibiotic-resistant phenotype that poses a serious clinical challenge. Particularly, the emergence of extended-spectrum β-lactamases (ESBLs) and carbapenemases resistant *P. mirabilis* strains. On a molecular level, *P. mirabilis* possesses a wide array of virulence factors including the production of fimbriae, urease, hemolysins, metallophores, and biofilm formation. This review thoroughly tackles a substantial gap in understanding the role of metallophores in shaping the virulence factors of *P. mirabilis* virulence. Siderophores, iron metal chelating and transporting metallophores, particularly contribute to the complex pathogenic strategies, displaying a potential target for therapeutic intervention.

## 1 Introduction to *Proteus mirabilis*: an overview of its molecular and biological features

### 1.1 General characteristics

*Proteus mirabilis (P. mirabilis)* is a Gram-negative bacterium, that has facultative anaerobic characteristics, and a rod-shaped morphology. Classified within the *Gammaproteobacteria* class, it has long been acknowledged as a member of the *Enterobacteriales* order and *Enterobacteriaceae* family (Adeolu et al., 2016). Initially, this motile bacterium has been identified by Gustav Hauser in 1885 (Drzewiecka, 2016). Its notable urease production, distinctive “swarming” behavior on agar plates, and rapid and coordinated multicellular activity were the first characteristics highlighted by Gustav Hauser. Its motility is facilitated by peritrichous flagella, essential for its movement across surfaces and resulting in a recognizable “bull’s-eye” pattern (Zabłotni et al., 2018). The sequencing of the bacterium’s genome in the early 21st century has offered insights into its metabolic adaptability in various environments. Notably, *P. mirabilis* can be found in diverse settings, including soil, water, and sewage, where it plays a key role in organic matter decomposition (Armbruster et al., 2018). However, it is considered an integral part of the fecal flora and hence can be mainly found in the digestive systems of humans and animals (Wenner and Rettger, 1919; Armbruster and Mobley, 2012). Although it can accommodate different environments, the bacterium exhibits optimal proliferative activity at temperatures ranging from 34 to 37°C, rendering the human body as an optimal habitat (Armbruster and Mobley, 2012). Moreover, its distinct swarming motility, coupled with its capacity to undergo auto-elongation and excrete polysaccharides upon interfacing with solid substrates, facilitates both adherence and efficient translocation across biomedical devices such as catheters, intravenous tubing, and other clinical apparatuses (Marcon et al., 2019; Jamil et al., 2023).

### 1.2 *P. mirabilis–*The disease: epidemiology, risk factors, and characteristics

Remarkably, *P. mirabilis* can lead to a range of human infections encompassing wounds, ocular regions, gastrointestinal tract, and the urinary system (Govindarajan and Kandaswamy, 2022). *P. mirabilis* is particularly recognized for its association with infections in the catheterized urinary tract, termed catheter-associated urinary tract infections (CAUTIs), with the highest incidence in elderly patients (Armbruster et al., 2018; Werneburg, 2022). It causes urinary tract infections (UTIs) with a higher incidence rate in the 20 to 50-year age range, particularly among females (Medina and Castillo-Pino, 2019). *P. mirabilis* is responsible for 1–2% of UTI cases among healthy adult females, whereas it is responsible for 5% of nosocomial UTI cases among hospitalized females. Additionally, the incidence of *Proteus*-related UTIs rises significantly in cases of complicated UTIs, for instance following urinary catheterization the incidence rate may rise to 45% (Jamil et al., 2023).

Risk factors for the development of *proteus*-mediated UTIs include sexual activity in both genders, with additional specific risks accompanied by unprotected anal intercourse between males and the presence of the foreskin. Also, immunocompromised states such as a decrease in CD4 cell count to fewer than 200 cells per microliter, elevate UTI risk. Furthermore, the likelihood of *P. mirabilis* infection is amplified in females with prolonged catheterization, inadequate catheter sanitation or maintenance, pre-existing medical conditions, and the unavailability of systemic antibiotic therapies (Jamil et al., 2023). In general, indwelling urinary catheters are categorized as short-duration if they are used for fewer than 30 days and chronic or long-duration if they are used for more than 30 days (Rubi et al., 2022). The primary mode of transmission for this bacterium involves direct contact with infected individuals or exposure to contaminated surfaces. Its remarkable motility supports quick dissemination, enabling it to enter the human urogenital system from the intestine. This entry is facilitated through the ingestion of contaminated food or via smear infection (Proteus mirabilis, 2024). Once within the gastrointestinal tract *P. mirabilis* can move to the urinary system and initiate UTIs, as evidenced by its simultaneous presence in both urinary and fecal samples (Proteus mirabilis, 2024). Alternatively, the bacterium may propagate through nosocomial pathways in instances where the gastrointestinal flora does not serve as a reservoir (Proteus mirabilis, 2024). Since it is most frequently associated with infections of UTIs, especially in complicated or CAUTIs, it is noted that these infections are most manifested in patients with prolonged catheterization. Including but not limited to patients residing in nursing facilities and long-term care institutions (Hung et al., 2007).

Patients harboring a *Proteus* infection may manifest clinical symptoms consistent with urethritis, cystitis, prostatitis, or pyelonephritis. A medical record revealing recurrent nephrolithiasis could suggest a persistent *Proteus* infection, given the fact that a stone formation history may indicate chronic *Proteus* infection (Jamil et al., 2023). These bacteria generate urease, a bacterial enzyme that is essential to the crystallization process and one of the primary indicators of the bacteria’s pathogenicity. The crystals of struvite and amorphous carbonate apatite are the most often occurring solid components of pathogenic urinary stones. There are two primary mechanisms involved in the production of these stones. The nucleation of solid phases is the first step, and the agglomeration of crystalline and amorphous precipitated phases is the second (Prywer and Olszynski, 2017). *P. mirabilis-*instigated UTIs and CAUTIs can also be exacerbated by the development of urolithiasis in the bladder and kidneys, potentially causing irreversible renal impairment (Griffith et al., 1976; Foxman and Brown, 2003; Armbruster et al., 2018). *P. mirabilis*-caused UTIs can further progress into bacteremia and sepsis, whether by damaging the renal parenchyma or by utilizing catheters as “mediators” allowing them to move along surfaces and spread across tissues (Chen et al., 2012; Jamil et al., 2023). *P. mirabilis* is frequently identified as a common causative agent for Gram-negative bacteremia, especially in patients with coexisting UTIs. Recent studies have reported the detection of *P. mirabilis* in 5–18% of Gram-negative bacteremia cases (Lubart et al., 2011). Notably, an elevated mortality rate of up to 50% has been observed in elderly cohorts diagnosed with this condition (Lubart et al., 2011).

### 1.3 Diagnosis and treatment

The most conclusive method for *P. mirabilis* diagnosis is culturing the bacterium after its isolation from infection sites or affected organs (Proteus mirabilis, 2009). Typically, the majority of *P. mirabilis* strains demonstrate a lack of lactose fermentation and a distinctive swarming motility. These distinctive characteristics are readily discernable on agar plates, facilitating the efficient identification of *P. mirabilis* (Jamil et al., 2023). From a clinical perspective, *P. mirabilis* infection may manifest in symptoms such as dysuria, marked by pain or burning sensations during urination, turbid urine, increased urinary frequency, abdominal discomfort, and systemic indicators of infection such as fever and chills. Empirical treatment of uncomplicated *P. mirabilis* infections typically mirrors that of other uncomplicated bacterial UTIs, favoring outpatient treatment modalities. This commonly involves a 3-day course of trimethoprim/sulfamethoxazole (TMP/SMX) or an oral fluoroquinolone, such as ciprofloxacin (Armbruster et al., 2018; Sabih and Leslie, 2024). In the case of acute, uncomplicated pyelonephritis, outpatient therapy also utilizes fluoroquinolones; however, a prolonged course ranging from 7 to 14 days is advisable (Sabih and Leslie, 2024). An alternative therapeutic strategy comprises an initial single dose of ceftriaxone or gentamycin, followed by a 7 to 14-day course of TMP/SMX, an oral fluoroquinolone, or a cephalosporin (Sabih and Leslie, 2024). In cases where patients demonstrate severe UTI symptoms, demanding an inpatient setting, the initiation of intravenous antibiotics is recommended. Preferred agents may include ceftriaxone, gentamycin, fluoroquinolone, gentamycin plus ampicillin, or aztreonam. Treatment must continue until the patient reaches an afebrile status. Afterward, a transition to oral therapy with cephalosporins, an oral fluoroquinolone, or TMP/SMX for an additional period of up to 14 days is recommended (Sabih and Leslie, 2024). Patients with complicated UTIs, such as those with predisposing underlying conditions that may potentiate treatment failure, may also undergo outpatient treatment. This treatment would involve oral antibiotics for an extended duration of 10 to 21 days, coupled with diligent follow-up care. In healthy immunocompetent individuals, symptoms of *P. mirabilis* infection typically resolve without lasting consequences (Armbruster et al., 2018; Jamil et al., 2023; Sabih and Leslie, 2024). Conversely, immunocompromised patients are at an elevated risk of developing severe complications such as sepsis or protracted infections, necessitating vigilant medical oversight. Preventive measures against *Proteus* infection are pivotal and include stringent sanitation and hygiene practices. These measures encompass the proper sterilization of medical instruments and environmental surfaces. Moreover, the utilization of catheterization should be practiced cautiously, being reserved exclusively for those patients for whom alternative options are not feasible (Armbruster et al., 2018; Sabih and Leslie, 2024).

### 1.4 *P. mirabilis*–Associated antibiotics resistance

On the other hand, the analysis of *P. mirabilis* genome has revealed numerous genes associated with antibiotic resistance (Adamus-Bialek et al., 2013). This finding is consistent with the growing clinical challenge of treating *P. mirabilis* infections, due to its resistance to commonly used antibiotics such as imipenem, cephalosporins, penicillins, and aztreonam (Foxman and Brown, 2003). *P. mirabilis* and *P. vulgaris* inherently exhibit resistance to a spectrum of antimicrobial agents, including polymyxins (notably colistin), nitrofurantoin, tigecycline, and tetracyclines. A notable characteristic of *P. mirabilis* is its lack of chromosomally encoded β-lactamases, rendering the wild-type phenotype fully susceptible to all β-lactam antibiotics (Girlich et al., 2020). However, within healthcare settings, the incidence of *P. mirabilis* strains resistant to amoxicillin is reported to be comparable to that of *Escherichia coli* (*E. coli*), with prevalence rates ranging from 38 to 48.5% (Yong et al., 2006; Girlich et al., 2020). This underscores the need for careful antibiotic stewardship and monitoring of resistance patterns in clinical environments.

Extended-spectrum β-lactamases (ESBLs), first reported in 1983, and plasmid-mediated AmpC β-lactamases, reported in 1988, are mutated, plasmid-borne enzymes evolved from broad-spectrum β-lactamases such as TEM-1, TEM-2, and SHV-1 (Girlich et al., 2020). These enzymes have broadened their hydrolytic activity, enabling the breakdown of extended-spectrum cephalosporins, penicillins, and aztreonam. While commonly produced by *Klebsiella* spp. and *E. coli*, these enzymes can also be found in other Gram-negative bacteria, including those from the *Morganellaceae* family. In *P. mirabilis*, various resistance genes have been identified, encoding for narrow-spectrum β-lactamases like TEM, SHV, CARB, and IRT derivatives, as well as acquired cephalosporinases (e.g., DHA, CMY, ACC-1), ESBL types (e.g., TEM/SHV, CTX-M, VEB, PER), and carbapenemases. Epidemiological studies indicate a significant rise in ESBL-producing *P. mirabilis* isolates. For instance, the prevalence of ESBL-producing *P. mirabilis* in Taiwan increased threefold within 4 years. Similarly, a high rate of ESBL production was observed in *P. mirabilis* isolates from India, with a considerable percentage also co-producing AmpC and carbapenemase (Datta et al., 2014). While the prevalence of carbapenemase-producing *P. mirabilis* remains low globally, it has shown a tendency to increase. Cases of KPC-2 carbapenemase-producing *P. mirabilis* have been reported in various countries across the globe (Girlich et al., 2020). Furthermore, the identification of resistance genes in *P. mirabilis* against additional antibiotic classes like quinolones and aminoglycosides is continuously growing. These genes are commonly situated on mobile genetic elements, encompassing plasmids, transposons, genomic islands, and integrons, facilitating their spread through horizontal gene transfer (Girlich et al., 2020).

### 1.5 Virulence characteristics

On a molecular level, *P. mirabilis* possesses a diverse array of virulence factors that enhance its colonization and pathogenic capabilities. Notably, fimbriae play a crucial role in facilitating adhesion to uroepithelial cells, while the urease enzyme contributes to the breakdown of urea into ammonia and carbon dioxide (Armbruster et al., 2018). Additionally, hemolysins are instrumental in damaging host cell membranes, further heightening its virulent nature. The interplay of these factors underscores its significance in the development of CAUTIs and the formation of struvite stones, establishing it as a prominent uro-pathogen. Moreover, the formation of biofilms represents a captivating facet of *P. mirabilis* biology. These biofilm communities, composed of bacteria enclosed within a self-produced matrix, confer protection against antimicrobial agents and host immune responses. This presents challenges in clinical treatment, highlighting the bacterium’s adaptive strategies for persistence and pathogenicity (Armbruster et al., 2018). The *P. mirabilis* exhibits a sophisticated virulence mechanism by adeptly utilizing metallophores, contributing to the complex orchestration of its pathogenic strategies. In this context, exploring the role of metallophores in shaping these virulence factors becomes crucial, as these small molecules involved in metal chelation and transport may add a layer of complexity to the pathogenic strategies of *P. mirabilis* (Armbruster and Mobley, 2012; Armbruster et al., 2018).

This review aims to provide a comprehensive exploration of the role of metallophores in shaping the virulence factors of *P. mirabilis*. While there is an existing body of knowledge on the virulence mechanisms of *P. mirabilis*, there remains a significant gap in understanding how metallophores specifically contribute to the formation and modulation of these factors. Metallophores, being small molecules involved in metal chelation and transport, have been implicated in various bacterial pathogenic processes (Armbruster et al., 2018). However, their specific involvement in the context of *P. mirabilis* and its virulence factors is a domain that demands closer examination. By addressing this gap, the review aims to not only enhance our comprehension of the intricate molecular strategies employed by *P. mirabilis* for pathogenicity but also to provide potential insights for the development of targeted therapeutic interventions.

## 2 A scope into *P. mirabilis* virulence factors

*Proteus mirabilis* exhibits sophisticated molecular mechanisms that efficiently enhance its virulence. One of these mechanisms is its distinctive swarming motility, characterized by coordinated flagellar movement. This unique motility enhances its colonization and persistence in diverse host environments. Another key virulence factor is urease which hydrolyzes urea, generating ammonia, elevating urinary pH, and contributing to alkaline urinary stone formation. Also, fimbrial adhesins further augment virulence by promoting adherence to host tissues. The bacterium also deploys a diverse array of secreted toxins and proteases, contributing to tissue damage and immune evasion. Understanding these molecular mechanisms is crucial for addressing the challenges posed by *P. mirabilis* in clinical management (Armbruster et al., 2018).

### 2.1 The swarming activity of *P. mirabilis*

**FIGURE 1:**
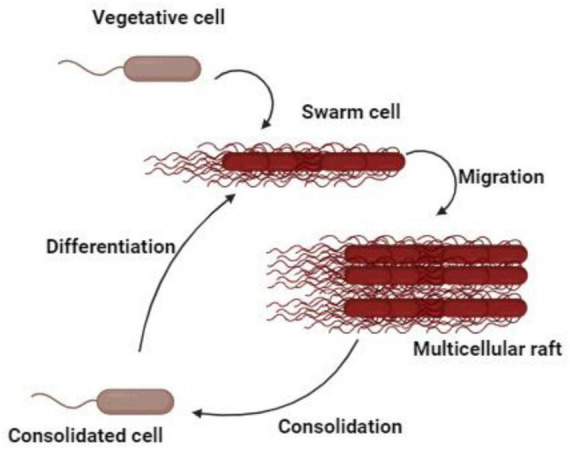
The differentiation process of swarming process.

*Proteus mirabilis* displays a form of multicellular behavior called swarming migration. This process encompasses the differentiation of vegetative cells at the colony margin into swarm cells. Swarm cells are elongated, aseptate, multinucleated, hyper-flagellated filaments that undergo repeated cycles of coordinated population merging and migration. The swarming activity of *P. mirabilis* is a multifaceted and dynamic process characterized by unique cellular behaviors (Williams and Schwarzhoff, 1978). During the formation of swarm cells, DNA replication occurs without septation, resulting in the development of elongated, polyploid cells (Williams and Schwarzhoff, 1978). At distinct intervals, these bacteria momentarily cease their movement, undergoing a process known as consolidation, which transitions them into a shorter morphotype (Gué et al., 2001). Subsequently, they re-differentiate into swarm cells, distinguished by their remarkable length (typically 20–80 μm) and hyper-flagellation (Gué et al., 2001; Strating et al., 2012). The expression of flagella is significantly upregulated during the initial transition from swimming to swarming and persists regularly throughout the swarm cycle (Figure 1). The coordinated movement of swarmer cells produces a distinctive *bull’s-eye* pattern on various media surfaces, with cells moving as rafts of parallel entities. Capsular polysaccharides, known as colony migration factor, and an uncharacterized slime are associated with swarming cells, which may aid in movement across surfaces (Gué et al., 2001). Remarkably, *P. mirabilis* exhibits an exceptional ability to swarm across catheters made of silicone or latex, posing risks for catheterized patients (Stickler and Hughes, 1999). Furthermore, the elevated expression of virulence genes during swarming suggests a potential role in UTIs, though their exact contribution remains a matter of debate (Allison et al., 1994). Additionally, flagella play a pivotal role in the swarming behavior, utilized by *P. mirabilis* for swimming in liquids and chemotaxis. In liquid culture, the bacterium adopts a short rod shape with peritrichous flagella. However, on solid media, it differentiates into long, non-septate, polyploid cells with numerous flagella facilitating collective surface movement (Stahl et al., 1983; Stickler and Hughes, 1999). Beyond flagellar components, non-flagellar elements such as lipopolysaccharide (LPS), capsule, and cell morphology also contribute to the regulation of swarming. Changes in LPS, peptidoglycans, and membrane fatty acids composition accompany the shift to the swarmer form. The association of capsular polysaccharides and an unidentified slime with swarming cells further emphasizes their role in surface motility (Rahman et al., 1999). The complex nature of this phenomena is demonstrated by differential gene regulation during swarming, which includes genes that are not strictly necessary for the swarming process (Allison et al., 1993). Despite sharing swarming features with other bacterial species, *P. mirabilis* is distinguished by its unique and robust swarming. This occurs on most laboratory media unless specific inhibitors are employed and is notably absent on chemically defined minimal media.

The swarming activity of *P. mirabilis* induces key virulence factors, enhancing its virulence compared to older bacteria or broth culture within a swarm colony. Notably, virulence factors such as urease, *ZapA* protease, and hemolysin exhibit increased expression levels during swarming, highlighting the dynamic nature of this process (Allison et al., 1992; Fraser et al., 2002). The regulation of virulence factors is intricately linked to the expression of specific genes and the activity of regulatory proteins. Overexpression of the flagellar regulator *umoB* results in an elevation of both *ZapA* and *HpmA*, which are hemolysin production genes, emphasizing the interplay between flagellar regulation and virulence factor expression. Additionally, the transcription of *hpmA* is responsive to *Lrp*, adding another layer of complexity to the regulatory network governing swarming and virulence (Fraser et al., 2002).

The clinical relevance of *P. mirabilis* swarming is underscored by its ability to traverse catheters made of silicone or latex, making it especially pertinent to catheterized patients (Sabbuba et al., 2002; Jones et al., 2004). This swarming behavior on catheters may be linked to the increased expression of virulence genes, raising the possibility of UTI initiation during catheterization. Studies involving intravenous infection of outbred mice with *P. mirabilis* demonstrated extensive kidney infection, with long-form swarmer bacteria observed in kidney parenchymal tissue 15 days post-infection (Allison et al., 1994). The manifestation of swarming might be dependent on the presence of a catheter or during the potential invasion of renal cells at later stages of disease progression (Lubart et al., 2011). The intricate relationship between swarming behavior and metabolic pathways further adds to the complexity of *P. mirabilis*’s virulence. The putrescine biosynthetic pathway, which is crucial for initiating swarming, is linked to urea formation (Pearson et al., 2008). The presence of genes capable of catalyzing the generation of putrescine and urea from ammonia and ATP suggests a potential regulatory role for these metabolites during swarming behavior (Pearson et al., 2008). Interestingly, the abundant urea in the urinary tract may act as a regulatory factor, repressing swarming behavior by influencing the putrescine biosynthetic pathway in the opposite direction.

#### 2.1.1 Important components contributing to *P. mirabilis* swarming activity

##### 2.1.1.1 Flagella

The role of flagella in the swarming activity and virulence of *P. mirabilis* is integral and complicated. Transcription of flagella is substantially increased during the initial transition from swimming to swarming, maintaining a high cyclic expression throughout the swarm cycle. Microarray analysis measuring the transcription of swarm and consolidated cells identified *flaA (flagellin)* as the third and sixth most highly expressed transcripts, respectively (Pearson et al., 2010). This highlights the crucial role of flagella in both swarming motility and swarming cell differentiation. The sensory function of flagella in detecting solid or viscous surfaces is proposed to be pivotal in initiating swarming behavior. It is hypothesized that the restricted rotation of flagella on such surfaces transmits signals to trigger the expression of swarming associated genes (Allison et al., 1993; Gygi et al., 1995; Belas and Suvanasuthi, 2005). This mechanistic insight underlines the significance of flagellar sensing in coordinating the swarming response. The impact of mutations on flagellar genes is evident in their effect on motility, swarm, and elongation, resulting in a wild-type phenotype (Gygi et al., 1995; Belas and Suvanasuthi, 2005). This aligns with the hypothesis that surface sensing by flagella is a prerequisite for swarming differentiation. The interconnection of flagellar function with motility and swarming further highlights the central role of flagella in orchestrating the complex behaviors associated with *P. mirabilis’s* swarming activity. Swarming and swimming are two flagellum-dependent *P. mirabilis* motilities that aid in the development of biofilms and the spread of infection. Additionally, there is a correlation between the development of hyperflagellated swarmer cells and notable elevations in the virulence factors such as urease and protease. Given that non-motile bacteria create fewer biofilms than motile ones, a strong relationship between biofilm development and bacterial motility has been proposed. Therefore, in addition to its motility, the capacity of bacteria to form biofilms leads to the spread of infection and an increase in the antibiotic resistance of the persistent germs (Khayyat et al., 2021).

##### 2.1.1.2 Capsule and cell morphology

The swarming activity and virulence of *P. mirabilis* are closely related to the morphology of its cells and capsules. Mutations in capsule-related genes and perturbations in zinc and iron disposition exert profound effects on *P. mirabilis* swarming behavior. Specifically, a mutation in the colony migration factor gene, *cmfA* (PMI3190), results in a capsular polysaccharide defect, leading to the emergence of elongated and hyperflagellated cells with a concomitant reduction in swarm velocity (Hay et al., 1999). The importance of this capsular polysaccharide is underscored by the determination of its tetrasaccharide repeat structure, which significantly contributes to the overall virulence of *P. mirabilis* (Allison et al., 1994). Another key player in cell morphology and swarming differentiation is the curved cell morphology gene, *ccmA* (PMI1961) (Schaffer and Pearson, 2015). Transposon mutants with motility and elongation-positive but swarming-deficient phenotypes have been localized to *ccmA*. These mutants exhibit hyperflagellated and elongated cells, characterized by a distinctive curved and uneven-width phenotype. Overexpression of *ccmA* results in cells with ellipsoidal or spherical shapes, suggesting its role in maintaining linearity during swarm cell elongation. As an integral membrane protein, *ccmA’s* expression increases during swarming differentiation, and it is implicated in organizing peptidoglycan assembly (Pearson et al., 2010; Schaffer and Pearson, 2015). Furthermore, a regulatory link between LPS biosynthesis and swarming differentiation is established (Schaffer and Pearson, 2015). Mutations in LPS biosynthesis genes; *rcsB* or *rcsC*, and to a lesser extent *rcsF*, suppresses the swarming deficiency in the *waaL* mutants. Highlighting an intricate relationship between LPS and swarming in *P. mirabilis* (Allison and Hughes, 1991; Morgenstein and Rather, 2012). These findings provide valuable insights into the molecular mechanisms governing the pathogenicity of this bacterium.

##### 2.1.1.3 Zinc and iron acquisition

The virulence and swarming activity of *P. mirabilis* depends on capsule formation, zinc acquisition, and iron utilization. A study by Lai et al. (1998) reveals the impact of zinc transport on swarming, in which disrupting the membrane P-type ATPase zinc-transporter gene (PMI3600 *ppaA*), leads to the formation of an abnormal swarm mutant (Rensing et al., 1998; Himpsl et al., 2008). This mutant displays diminished swarm velocity, incomplete elongation, prolonged swarming intervals, and abnormal consolidation terracing. Despite lower levels of flagellin transcript and protein production, along with the repression of the motility regulator *lrp*, the mutant maintains normal swimming motility. Remarkably, during swarming in wild-type *P. mirabilis*, the expression of ppaA is induced, highlighting its importance in the swarming process (Hay et al., 1997; Rensing et al., 1998). Additionally, the complex network of genes contributing to swarming in *P. mirabilis* has been identified through two signature-tagged mutagenesis (STM) screens (Burall et al., 2004; Armbruster et al., 2013). However, the specific roles of these genes in the swarming process remain to be fully elucidated, leaving a gap in our understanding of the molecular mechanisms governing swarming activity.

##### 2.1.1.4 Extracellular contributors to swarming

The initiation of swarming in *P. mirabilis* involves a complex interplay of extracellular contributors, shedding light on the diverse molecular signals that drive this phenomenon. A study by Allison et al. (1993) demonstrated that only glutamine, among various components added to a defined minimal growth medium, is capable of triggering swarming behavior. This finding underlines the specific and essential role of glutamine in the induction of swarming. To expand the understanding of swarming signals, recent research by an undisclosed study utilized a rich medium (LB) with low NaCl concentration (10 mM) to identify additional factors contributing to swarming in *P. mirabilis* HI4320 (Schaffer and Pearson, 2015). Under these conditions, swarming at 37°C was not observed. However, the addition of 20 mM of *L-glutamine*, *L-arginine*, *DL-histidine*, *malate*, *or DL-ornithine* effectively promoted swarming, emphasizing the multifaceted nature of extracellular contributors to this bacterial behavior (Armbruster et al., 2013). Another significant external factor is putrescine, identified as a potential inducer of differentiation in *P. mirabilis* (Armbruster et al., 2013). The persistent generation and buildup of putrescine in the growth medium, along with its existence in the outer membrane of specific Gram-negative bacteria including *P. mirabilis*, underscore its role in the initiation of swarming (Sturgill and Rather, 2004; Armbruster et al., 2013).

### 2.2 Fimbrea

The exploration of *P. mirabilis* at the genomic level has revealed a spectrum of adhesive structures extending beyond the extensively studied chaperone-usher fimbriae. Notably, the bacterium’s genome harbors genes encoding one or two potential type IV pili. This indicates that the bacteria exhibit a diverse range of adhesins which play a significant role in its ability to cause disease (Silverblatt, 1974; Kuan et al., 2014). In bacteria, type IV pili are multi-subunit molecules that resemble hair and are essential for pathogenicity, colonization, twitching motility, and the production of biofilms. These pili exhibit special characteristics such as pilin polymerization and depolymerization, which cause the pili to lengthen and shorten, respectively. It is made up of helix-shaped polymer copies of one or more pilin subunits (Persat et al., 2015). Type IV pili is made up of multiple pilin subunits, each serving a distinct function. These subunits included major and minor pilins, pre-pilin peptidase, specific ATPase that functions as a motor and supplies energy for pilus assembly, inner membrane protein that recruits ATPase from the cytoplasm, outer membrane secretory protein that secretes pili proteins, inner membrane accessory proteins, and the retraction ATPase that encourages the depolymerization of pilus fiber known as twitching motility (Shanmugasundarasamy et al., 2022). The identification of a putative type IV pilus on the surface of *P. mirabilis* is noteworthy, with its size and expression pattern aligning closely with the MR/P fimbria, it emphasizes the complex web of adhesive mechanisms employed by this bacterium (Pearson et al., 2008; Armbruster et al., 2018). Fimbriae play a pivotal role in the virulence of *P. mirabilis*, serving as key adhesion structures that facilitate the bacterium’s interaction with host tissues. The chaperone-usher fimbriae, characterized by their molecular specificity, enable *P. mirabilis* to adhere to and colonize various surfaces within the host, including urinary tract epithelial cells (Armbruster et al., 2018). These adhesive structures contribute to the establishment of biofilms, aiding in the evasion of host immune responses and promoting persistent infections. Additionally, fimbriae assist in the formation of crystalline biofilms and encrustation, which are hallmark features of *P. mirabilis*-associated UTIs. The orchestrated role of fimbriae in adhesion and biofilm formation underscores their significance in *P. mirabilis* pathogenicity (Armbruster et al., 2018). Bacteria in a biofilm can coordinate their actions through a communication mechanism known as quorum sensing (QS). A key function of quorum sensing (QS) is to control the synthesis of virulence factors. Simply, QS refers to the bacterial population’s communication system. To change the expression of virulence proteins, certain QS receptors can detect their homologous inducers produced by the same bacterial species or even by other species (Elfaky et al., 2023). Despite using different inducers and QS machinery, both Gram-positive and Gram-negative bacteria use QS to modulate their pathogenicity (Thabit et al., 2022). QS is responsible for coordinating the development of numerous virulence factors, including biofilm formation, bacterial motility, and the synthesis of enzymes such as urease, elastase, protease, hemolysins, and other virulent factors. This system is triggered when the bacterial population reaches a threshold, at which point the biofilm’s constituents launch a coordinated group response in the community’s best interests, with the goal of preserving nutrients and energy by lowering the metabolic activity of the biofilm’s inhabitants. Furthermore, bacterial gene expression is focused on producing more virulence factors, particularly extracellular toxins, which cause significant tissue destruction at the biofilm site. This promotes the spread of infection to nearby tissues, further solidifying the biofilm and lengthening its persistence, and also allows for the generous release of nutrients from the damaged tissues (Cavalu et al., 2022; Lila et al., 2023; Rajab and Hegazy, 2023).

### 2.3 Hemolysin

*Proteus mirabilis* employs various strategies to evade host defenses, including the expression of a Serratia-type calcium-independent hemolysin, encoded by the *hpmA* gene. This hemolysin exhibits the capacity to lyse both nucleated cells and enucleated red blood cells, highlighting its broad cytolytic activity (Swihart and Welch, 1990; Uphoff and Welch, 1990; Cestari et al., 2013). Additionally, the membrane transporter responsible for the secretion of hemolysin is encoded by *hpmB* and exhibits remarkable conservation among *P. mirabilis* isolates emphasizing the pivotal role of this hemolytic system in the bacterium’s pathogenicity (Fraser et al., 2002). This produced cytolytic toxin plays a pivotal role in the invasion of host tissues and the initiation of infections. Its ability to lyse red blood cells is noteworthy, as it not only releases essential nutrients from host cells but also creates a favorable niche for the rapid proliferation of the bacterium within the host environment (Fraser et al., 2002).

Research findings indicate that hemolysin-deficient *P. mirabilis* mutants display a diminished capacity for virulence, highlighting the importance of this toxin in the bacterium’s overall pathogenicity (Alamuri et al., 2010; Armbruster et al., 2018). Beyond its role in nutrient acquisition and tissue invasion, hemolysin also plays a crucial part in immune evasion. By disrupting host cell membranes, hemolysin facilitates the spread of *P. mirabilis* within the host, allowing the bacteria to evade immune cells. Thus, contributing to the severity and persistence of infections. In essence, the multifaceted functions of hemolysin highlight its importance as a key virulence factor in the pathogenic strategy of *P. mirabilis* (Norsworthy and Pearson, 2017).

### 2.4 Proteases

Proteases are not only essential for the virulence of *P. mirabilis* but also for its survival, especially in the urinary tract. The environment within the urinary tract is challenging for the bacteria as it faces strong host defenses like antibodies and antimicrobial peptides. Among the various proteases utilized by *P. mirabilis*, *ZapA* (mirabilysin) is a potent metalloprotease capable of efficiently degrading numerous host proteins *in vitro* (Walker et al., 1999; Belas et al., 2004). The identification of potent chemical inhibitors for *ZapA* flags it as a potential therapeutic target. This discovery establishes a basis for future studies focused on disrupting this essential virulence factor, offering a potential avenue for mitigating *P. mirabilis* infections (Belas et al., 2004). *P. mirabilis* pathogenesis involves proteases beyond adhesion and hemolysis. *ZapA*’s ability to degrade a diverse array of host proteins *in vitro* underscores its adaptability and emphasizes its significance in disrupting the host immune response. As the pursuit of therapeutic interventions progresses, a deeper understanding of the regulatory mechanisms governing these virulence factors becomes crucial for developing targeted strategies to effectively combat *P. mirabilis* infections (Belas et al., 2004).

Remarkably, *ZapA* is induced during swarming differentiation, and its expression remains high at the edge of an expanding swarm colony (Allison et al., 1992; Phan et al., 2008). Furthermore, in a rat model of infection, *ZapA* contributes to acute prostatitis, and a *ZapA* mutant exhibits severe impairment in establishing chronic prostatitis in this model (Phan et al., 2008). Hence, *ZapA* plays a critical role in *P. mirabilis* pathogenesis, contributing to survival within the urinary tract and specific infection models. Therefore, it represents a potential effective target for therapeutic intervention.

### 2.5 Urease

*Proteus mirabilis* deploys a diverse array of virulence factors, with particular emphasis on the cytoplasmic nickel metalloenzyme “urease,” playing a pivotal role in pathogenesis (Griffith et al., 1976). Urease facilitates the enzymatic breakdown of urea into ammonia and carbon dioxide, leading to an elevation in local pH due to ammonia production (Kappaun et al., 2018). This alkaline pH triggers a cascade of events in the urinary tract, ending in the precipitation of calcium and magnesium ions and the formation of urinary stones, including magnesium ammonium phosphate (struvite) and calcium phosphate (apatite). The profound association between *P. mirabilis* virulence and urease activity is underscored by the consequential threat posed by urinary stones (Jones et al., 2005; Munns and Amawi, 2010; Chew et al., 2012). These stones can grow to considerable sizes and obstruct urinary flow, resulting in tissue damage (Li et al., 2002). The implications extend to urinary catheters, where precipitated minerals may combine with bacteria, forming a crystalline biofilm that gradually obstructs urine flow. This complex interplay creates a favorable environment for bacteria to embed within stones, affording protection against antibiotics and immune responses (Armbruster et al., 2014). Within the context of UTIs, the manifestation of urease activity extends beyond the influence of *P. mirabilis* alone. Instances of polymicrobial infections, exemplified by the experimental co-infection of mice with *P. mirabilis* and urease-positive *P. stuartii*, lead to an augmentation of urolithiasis and bacteremia (Armbruster et al., 2014). Despite comparable bacterial loads when compared to mono-species infection, the combined effects synergistically enhance the overall virulence potential (Armbruster et al., 2014). Recognizing the key role of urease in *P. mirabilis* virulence, ongoing investigations are actively directing their focus toward this enzyme for the development of clinically relevant inhibitors. Additionally, acknowledging that the bacterium’s capacity to generate urinary stones and crystalline biofilms hinges on an alkaline pH, an alternative strategy to prevent catheter blockage involves urine acidification. This comprehensive understanding of *P. mirabilis* virulence factors informs potential therapeutic strategies, emphasizing the importance of unraveling the mechanisms governing UTIs (Follmer, 2010).

**FIGURE 2:**
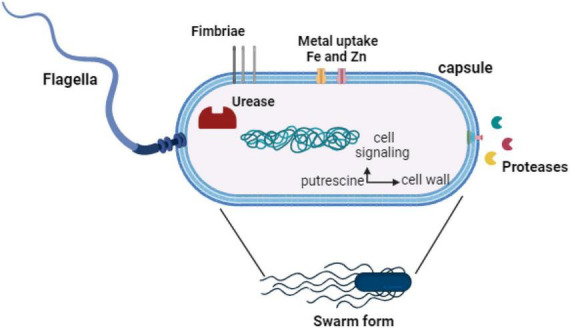
Virulence factors of *P. mirabilis.*

*Proteus mirabilis* employs a variety of virulence factors extending beyond the previously addressed swarming activity, fimbriae, hemolysin, proteases, and urease. These include adhesins, surface proteins expressed by *P. mirabilis*, which play a critical role in adhering to uroepithelial cells, a key step in UTIs (Danilo de Oliveira et al., 2021). This adherence mechanism enables the bacterium to withstand being washed away by urine flow, establishing a persistent infection. Furthermore, certain strains of *P. mirabilis* can generate protective capsules, providing a shield against the host immune system and bolstering resistance to phagocytosis (Beynon et al., 1992). Additionally, toxins produced by *P. mirabilis* further emphasize its pathogenicity. A comprehensive understanding of the diverse array of virulence factors employed by *P. mirabilis* allows researchers to investigate potential therapeutic targets and devise strategies to combat infections induced by this bacterium. Insights into these factors are pivotal for the development of effective treatments and vaccines aimed at mitigating the impact of diseases associated with *P. mirabilis*. Figure 2 summarizes the virulence factors of *P. mirabilis.*

## 3 Metal acquisition systems of *P. mirabilis*

*Proteus mirabilis* exhibits remarkable metal acquisition systems, illustrating microbial adaptation in challenging environments, especially in host-pathogen interactions. As a Gram-negative bacterium associated with UTIs, *P. mirabilis* has evolved complex mechanisms to acquire essential metals like iron, zinc, and manganese crucial for its survival and pathogenicity. In response to host defenses that regulate metal availability, *P. mirabilis* employs sophisticated metal acquisition systems, particularly for iron, a micronutrient often sequestered by the host (Cassat and Skaar, 2013). The bacterium uses siderophores to scavenge and solubilize iron in the extracellular environment, forming complexes selectively transported into the bacterial cell. Beyond iron, *P. mirabilis* has specialized transport systems for zinc, manganese, and nickel, ensuring optimal concentrations for metabolic processes. Understanding these systems not only reveals adaptive strategies but also suggests potential therapeutic interventions by targeting these mechanisms to disrupt bacterial survival, providing innovative approaches for treating *P. mirabilis* infections (Subashchandrabose and Mobley, 2015). Bacteria generally require approximately 10^–7^ to 10^–5^ M iron and 10^–15^ to 10^–16^ M zinc, respectively to achieve optimal growth. Mn^2+^ (total concentration of ∼2 μM) is imported by microorganisms for use in Mn-specific enzymes and can also be exchanged with iron in the metal-binding sites of some Fe proteins. Nickel (Ni), typically used by microbes for anaerobic growth, is found at <5 μM under aerobic conditions. Total Ni^2+^ in the cell may be increased under anaerobic growth when Ni-containing hydrogenase is expressed (Loutet et al., 2015).

### 3.1 Importing nickel for urease activity

**FIGURE 3:**
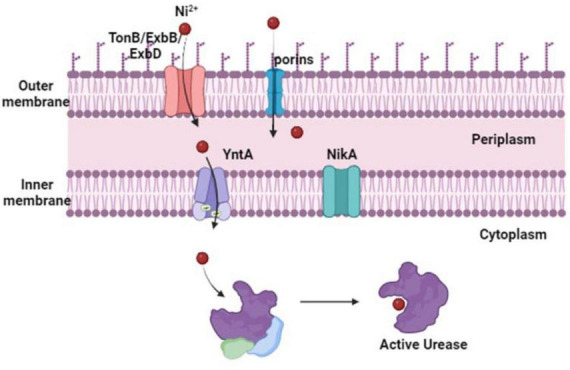
*Proteus mirabilis* nickel import routes for urease activity. The content of nickel in the urinary system is low. Although *P. mirabilis* import across the outer membrane has not yet been studied, it is most likely mediated by porins or the TonB/ExbB/ExbD machinery. After entering the periplasm, nickel is bound by a protein that binds to substrates and is moved by ABC transporters across the inner membrane to the urease and triggers its catalytic activation. Studies suggest that the Ynt transport system is typically used to do this. The Nik system can import and distribute nickel to the urease enzyme in the absence of YntA. But this only happens when there is a significant increase in the concentration of nickel.

*Proteus mirabilis* possesses a urease apoenzyme comprising three structural subunits, UreA, UreB, and UreC, assembled into a homotrimer of heterotrimers. However, for catalytic activity, each heterotrimer necessitates the incorporation of two nickel atoms (Heimer and Mobley, 2001). This incorporation process is facilitated by four accessory proteins encoded within the urease operon—UreD, UreE, UreF, and UreG. A total of six nickel atoms, two for each UreABC heterotrimer, result in the formation of a catalytically active holoenzyme (Brauer et al., 2020). Despite the importance of nickel in urease activation, its accumulation can lead to toxicity through various mechanisms, including mis-metalation, disruption of iron and copper homeostasis, and the generation of reactive oxygen species (Waldron and Robinson, 2009; Anjem and Imlay, 2012). To mitigate these risks, the transport of nickel is strictly regulated to ensure specific delivery to metalloproteins, rather than unrestricted release into the cytoplasm (Rowe et al., 2005; Zeer-Wanklyn and Zamble, 2017). Given that urease is in the cytoplasm of *P. mirabilis*, the transportation of nickel across the outer and inner membranes becomes imperative for its incorporation into the urease apoenzyme. Current understanding suggests that nickel transport across the outer membrane in Gram-negative bacteria is predominantly mediated by the TonB/ExbB/ExbD machinery, possibly assisted by porins (Li and Zamble, 2009). Once in the periplasm, nickel transport across the inner membrane is typically facilitated by dedicated ATP-binding cassette (ABC) transporters (Figure 3), such as the Nik system, or monomeric single-component permeases like the nickel/cobalt NiCoT permease (Li and Zamble, 2009). The transport machinery for nickel import is highly specific for delivery to specific metalloproteins, with ABC transporters primarily delivering nickel to urease and [NiFe]-hydrogenase (Boer et al., 2014). Besides, Nik and Ynt are two putative nickel import systems in the inner membrane, identified in the genome sequence of *P. mirabilis* strain HI4320 (Brauer et al., 2020).

#### 3.1.1 The importance of YntA and NikA to *P. mirabilis*

YntA serves as the primary nickel-binding protein crucial for urease activity in *P. mirabilis*. It has been established that the Ynt system is uniquely capable of delivering nickel to the urease enzyme, particularly under the concentrations found in human urine (Brauer et al., 2020). Although NikA has been identified as a compensatory mechanism in the absence of YntA, both Nik and Ynt are deemed as the exclusive nickel importers capable of delivering nickel for urease enzyme incorporation (Brauer et al., 2020). This conclusion is further supported by studies demonstrating the essential role of both Nik and Ynt systems in facilitating urease activity. Urease function is completely abolished in Ynt and Nik double mutants, urease activity was not rectified by excess nickel supplementation (Brauer et al., 2020). Notably, the significance of urease activity in *P. mirabilis* is underscored by its role as a critical virulence factor within the urinary tract. Loss of urease activity results in a pronounced defect in colonization and reduced pathology in experimental models of UTI (Jones et al., 1990; Johnson et al., 1993). Both the Nik and Ynt transport systems were upregulated during experimental UTI in mice. Moreover, in a model of CAUTI, Nik, and Ynt were recognized as contributors to *P. mirabilis* bladder and kidney colonization (Armbruster et al., 2017). These systems also emerged as infection-specific fitness factors for bloodstream infection, suggesting a potential role in dissemination or survival in the hosts’ bloodstream (Armbruster et al., 2019).

The importance of YntA and NikA to *P. mirabilis* fitness during infection is specifically attributed to their contribution to urease activity. Indeed, besides urease, *P. mirabilis* possesses other nickel-containing enzyme, [NiFe]-Hydrogenase, (Table 1) (Brauer et al., 2020). YntA is essential for both urease and hydrogenase activity, while NikA compensates for the loss of YntA, impacting the uptake of other metals indirectly. However, other nickel transporters are capable of supplying nickel to hydrogenase enzymes (Brauer et al., 2020). Both YntA and NikA contribute to fitness during CAUTI, with YntA providing the greatest advantage. The observed fitness defects in nickel transport mutants during CAUTI may stem from multiple factors, including urease and hydrogenase activities, as well as an impact on the transport of other metals (Brauer et al., 2020).

**TABLE 1:** Microbial nickel-containing enzymes and their functions.

Nickel-containing enzymes	Function
Glyoxalase I	The detoxification of methylglyoxal to prevent cellular component damage
Acireductone dioxygenase	Cleaves acireductone to formate, CO, and methylthiopropionate.
Urease	Urea hydrolysis into ammonia and carbamic acid
Superoxide dismutase	superoxide detoxification via converting it to O_2_ and H_2_O_2_
[NiFe]-Hydrogenase	Enables the utilization of molecular H_2_ as an electron donor in respiration and offers a way to get rid of extra reducing equivalents in fermentation.
Carbon monoxide dehydrogenase	Catalyzes the reversible oxidation of CO to CO_2_, thus it plays an important role in the global carbon cycle by enabling organisms to use CO as a source of carbon and energy
Acetyl-coenzyme A synthase/decarbonylase	CO_2_ fixation
Methyl-coenzyme M reductase	Methane metabolism
Lactate racemase	Converts substrate _D_-lactate into _L_-lactate.

### 3.2 Zinc importing system

**FIGURE 4:**
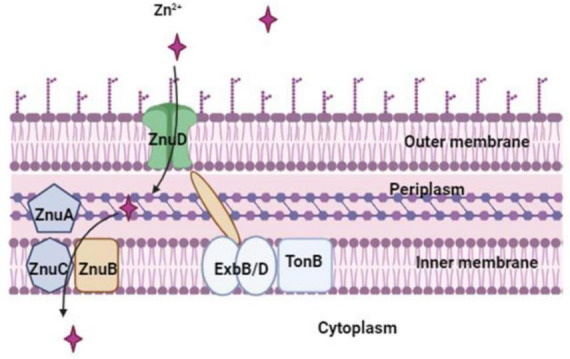
Zn^2+^ is imported by *P. mirabilis* via the Zn^2+^-regulated TonB-dependent ZnuD outer membrane receptor, which is powered by the Zn^2+^-regulated TonB-ExbB-ExbD system. The ZnuABC transporter, a member of the ABC transporter family, mediates zinc transport across the inner membrane.

*Proteus mirabilis* possesses a functional zinc uptake system (Figure 4), denoted as *znuACB* (Nielubowicz et al., 2010). This system becomes particularly significant in the context of UTIs, where zinc levels are believed to be limited. Acquiring zinc through the *znuACB* system provides *P. mirabilis* with a competitive edge against other pathogenic bacteria, including uropathogenic *E. coli* (Sabri et al., 2009). *In vivo*, the expression of *znuACB* in *P. mirabilis* is upregulated, and the absence of the *znuC* component results in a diminished ability of the mutant strain to compete with wild-type *P. mirabilis* during co-challenge infections (Nielubowicz et al., 2010; Pearson et al., 2011). This underscores the pivotal role of the *znuACB* system in *P. mirabilis* virulence. Certainly, Zinc is not only crucial for the metalloprotease ZapA, a factor known to confer a competitive advantage during infection but also for other virulence factors such as flagella. A *P. mirabilis znuC* mutant exhibits reduced flagellin expression, and since the master flagellar regulator (FlhD4C2) contains a zinc-binding site, zinc uptake might be essential for flagellar synthesis (Gaisser and Hughes, 1997; Wang et al., 2006). Considering the importance of zinc in regulating various virulence factors, including ZapA and flagella, the absence of a functional zinc-uptake system in *P. mirabilis* could also lead to improper regulation of gene expression (Nielubowicz et al., 2010; Armbruster et al., 2018). This deficiency may result in weakened colonization of the urinary tract due to compromised synthesis of flagellin, ZapA, and potentially other unidentified factors (Nielubowicz et al., 2010).

### 3.3 Iron importing system

Iron, an essential element, serves as a vital cofactor for numerous proteins and enzymes participating in diverse biological processes including oxygen transport, gene regulation, and nitrogen fixation. However, under aerobic conditions and neutral pH, iron predominantly exists in the potentially toxic ferric (Fe^3+^) form, which can become harmful when interacting with oxygen and oxygen-reduced species (Malik and Hedrich, 2022). Pathogens, including *P. mirabilis*, must overcome host iron sequestration during colonization to establish infections, as free iron is generally scarce *in vivo* (Himpsl et al., 2010). To counterbalance iron-limiting conditions and maintain iron homeostasis, bacteria, including *P. mirabilis*, have evolved a spectrum of iron transport systems, intracellular iron stores, redox stress resistance systems, and iron-responsive regulatory elements governing the expression of genes involved in various cellular functions (Lima et al., 2007). Notably, *P. mirabilis* has developed 21 iron acquisition systems employing a sophisticated repertoire of iron acquisition systems to thrive in iron-limiting conditions within the host environment (Lima et al., 2007; Schaffer and Pearson, 2015; Armbruster et al., 2018). These strategies include siderophore-based mechanisms, ferrous iron transport, metal-type ABC transporters, and other complex approaches (Lima et al., 2007). Understanding these systems is crucial for deciphering the pathogenicity of *P. mirabilis* and developing targeted interventions for urinary tract infections (Lima et al., 2007).

**FIGURE 5:**
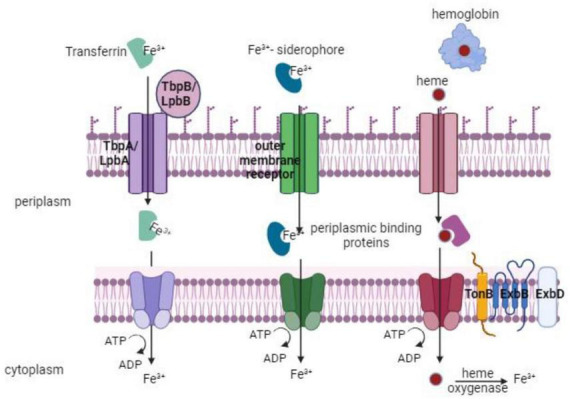
Iron take up by *P. mirabilis* via multiple routes, such as transferrin, siderophores, or heme uptake. An outer membrane receptor and an inner-membrane ABC transporter are necessary for each of these uptake pathways. The TonB system’s action is necessary for transport through the outer membrane receptor (TonB, ExbB, ExbD).

*Proteus mirabilis* mainly utilizes three ways to sequester iron: via proteobactin (Pbt), a non-ribosomal peptide synthesis system (Nrp), and α-keto acids (Armbruster et al., 2018). The *nrp* system was primarily defined as a signaling system regulated in response to iron during swarming development. However, analysis of the *P. mirabilis* genome revealed that the *nrp* together with proteobactin (pbt) are siderophore biosynthesis and transport operons (Armbruster et al., 2018) (discussed below in detail). *P. mirabilis* utilizes two amino acid deaminases (PMI2834 and PMI2149) to synthesize α-keto acids (Evanylo et al., 1984). These α-keto acids behave as non-classical siderophores and when supplied externally can promote the growth of *P. mirabilis* on iron-chelated media (Evanylo et al., 1984). Studies indicated that α-keto acids, produced by an amino acid deaminase (PMI2834), could effectively chelate iron from solutions (Drechsel et al., 1993; Massad et al., 1995). While α-keto acids appear to participate in iron chelation, current evidence suggests that they are likely not essential for growth during iron limitation (Drechsel et al., 1993). Furthermore, *P. mirabilis* cannot synthesize the siderophore enterobactin on its own but can utilize enterobactin produced by other enteric bacteria (Himpsl et al., 2010). Additionally, *P. mirabilis* utilizes ferric, ferrous, and heme uptake systems (Figure 5) that have been revealed by genetic and proteomic analysis during iron restriction, genome annotation, and transposon mutagenesis (Piccini et al., 1998; Lima et al., 2007; Ceccarelli et al., 2008; Himpsl et al., 2010). *P. mirabilis* demonstrates the ability to grow on hemin, with an alleged heme uptake system upregulated during iron limitation and *in vivo* conditions. The gene PMI1426 (hmuR2) encodes a putative outer membrane receptor in the heme uptake system. This receptor binds heme, is implicated in infection, and is identified as an antigen *in vivo* (Massad et al., 1995; Piccini et al., 1998; Himpsl et al., 2010). *P. mirabilis* also encodes genes predicted to participate in the uptake of ferrous iron, ferric iron, and ferric citrate. The repertoire also includes genes involved in iron-sulfur cluster formation and uptake, as well as several predicted TonB-dependent receptors and iron-related ABC transport systems (Piccini et al., 1998; Armbruster et al., 2018). Several of these genes have been identified as iron-regulated *in vitro* or have been found to contribute to UTIs by being positively regulated *in vivo*, expressed on the bacterial cell surface *in vivo*, or crucial for full colonization of the urinary tract (Armbruster et al., 2018).

Besides, under anaerobic and iron-limiting conditions, the ferrous iron transport system FeoAB, plays a crucial role in the uptake of ferrous iron (Fe^2+^) (Andrews et al., 2003). In conclusion, *P. mirabilis* exhibits versatility in iron utilization, encoding all four types of iron uptake systems and demonstrating the ability to grow on various iron sources, including hemoglobin, hemin, and ferric citrate. However, *P. mirabilis* cannot utilize transferrin and lactoferrin as iron sources (Piccini et al., 1998).

## 4 Unraveling *P. mirabilis* metallophores and their role in pathogenesis

Metallophores are small molecules synthesized by bacteria and serve as instrumental mediators in the acquisition of indispensable metal ions within their ecological medium. These compounds play a crucial role in diverse biological processes, mainly bacterial pathogenesis (Ghssein and Ezzeddine, 2022). *P. mirabilis* is another example of how metallophores are used in sophisticated ways by pathogens to promote their virulence. In the orchestration of pathogenesis, *P. mirabilis* strategically employs metallophores to sequester metal ions, particularly iron, from the host environment. Iron stands as a fundamental nutrient pivotal for bacterial growth and survival, yet its accessibility within the host is consistently restricted by the immune system’s modus operandi—iron sequestration, a defensive maneuver against bacterial incursions (Miethke and Marahiel, 2007). *P. mirabilis* selectively harnesses distinct metallophores, characterized by their diminutive size and heightened affinity for metal ions, to competitively engage and acquire iron from host tissues (Wassif et al., 1995). Siderophores are metallophores that exhibit the capacity to bind extracellular iron, facilitating its transportation into the bacterial cell, thereby ensuring a constant supply of this essential nutrient. Siderophores scavenge and transport ferric ions (Fe^3+^) via chelating iron ions to form stable complexes that are then taken up by microbial cells through specific receptors on their cell membranes. Such proficiency in iron acquisition through metallophores significantly amplifies the survival and propagation capabilities of *P. mirabilis* within the host medium. Furthermore, the synthesis of metallophores by *P. mirabilis* intricately intertwines with its virulence, as the efficacy of iron and other metals acquisition substantiates the bacterium’s proficiency in initiating and perpetuating infections (Wassif et al., 1995; Miethke and Marahiel, 2007). The competition for iron resources, intertwined with the host’s iron-binding proteins, is a key aspect of the microbial-host conflict race. Through effective iron acquisition facilitated by siderophores, *P. mirabilis* adeptly circumvents host immune defenses, establishing and sustaining a persistent infection (Miethke and Marahiel, 2007). Comprehending the multifaceted role of bacterial siderophores, particularly within the context of *P. mirabilis* pathogenesis, offers invaluable insights for formulating targeted therapeutic interventions. The prospect of inhibiting metallophore and siderophore production or functionality emerges as a promising therapeutic avenue. The potential disruption of the bacterium’s capacity to procure iron renders it more susceptible to host immune responses and antimicrobial treatments.

### 4.1 *P. Mirabilis* iron-sequestering metallophores: the *Siderophores*

**FIGURE 6:**
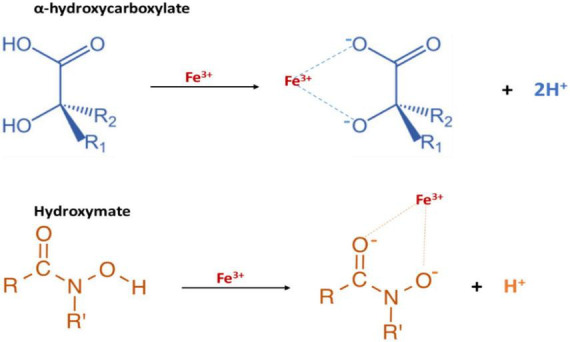
The functional groups of *P. mirabilis* Siderophores: proteobactin (hydroxy carboxylate siderophore) and yersiniabactin-related (mixed type hydroxymate and carboxylate siderophore).

*Proteus mirabilis* employs siderophores to secure essential iron in its pathogenic pursuits (Figure 6). These siderophores exhibit a remarkable capacity to scavenge extracellular iron from the host environment, enhancing the bacterium’s ability to thrive within the host (Miethke and Marahiel, 2007). The intricate utilization of siderophores by *P. mirabilis* exemplifies a strategic adaptation, allowing the bacterium to navigate the iron-limiting conditions imposed by the host’s immune defenses and contributing to its virulence in causing infections.

Indeed, the diversity of siderophores is extensive, with over 500 described, and they can be categorized into three groups: catecholates, hydroxamates, and hydroxy-carboxylates (Ahmed and Holmström, 2014). The precursors for siderophore biosynthesis in bacteria include citrate, amino acids, dihydroxybenzoate, and N^5^-acyl-N^5^-hydroxyornithine (Andrews et al., 2003). Notably, genes encoding the biosynthetic enzymes for siderophores are often clustered with those responsible for ferri-siderophore transport (Himpsl et al., 2010). Thus, the entire process of siderophore synthesis and transport is tightly regulated by the ferric uptake regulator (Fur) protein in response to iron availability (Himpsl et al., 2010). The siderophore effectively chelates ferric iron from the surrounding environment upon synthesis and secretion. Subsequently, the ferri-siderophore selectively binds to a TonB-dependent outer membrane receptor in Gram-negative bacterial envelopes (Braun, 1995; Neilands, 1995). The intracellular transport relies on the cytosolic membrane potential, facilitated by the TonB-ExbB-ExbD system, requiring direct contact between TonB and the TonB-dependent outer membrane receptor (Braun, 1995; Neilands, 1995). Once in the periplasmic space, a periplasmic binding protein targets the ferri-siderophore toward the membrane ABC transporter which facilitates its transportation into the cytoplasm. In the cytosol, the reduction of iron from ferric (Fe^3+^) to ferrous (Fe^2+^) triggers its dissociation from the siderophore (Miles and Khimji, 1975; Ratledge and Dover, 2000; Faraldo-Gómez and Sansom, 2003; Wandersman and Delepelaire, 2004).

Previous reports suggested the absence of detectable siderophore production in *P. mirabilis* (Miles and Khimji, 1975; Coker et al., 2000; Armbruster and Mobley, 2012). However, genomic analysis of *P. mirabilis* HI4320 revealed two gene clusters related to siderophore biosynthesis and the ABC transport system (Pearson et al., 2008). One of these clusters represents a novel non-ribosomal peptide synthetase (NRPS)-independent siderophore system named proteobactin (Himpsl et al., 2010). Another cluster contains the *nrp* operon is up-regulated during iron limitation and is located within the high-pathogenicity island (HPI) with homology to *Yersinia* spp. (Himpsl et al., 2010). Signature-tagged mutagenesis (STM) identified five genes associated with iron acquisition in *P. mirabilis* HI4320, including the putative TonB-dependent receptors, a 4′-phosphopantetheinyl transferase (nrpG), and a putative iron ABC transporter permease (Burall et al., 2004; Himpsl et al., 2008).

Both the *nrp* and proteobactin systems play roles in iron chelation, however, only *nrp* had evident implications in UTI (Himpsl et al., 2010). While wild-type *P. mirabilis* effectively chelates iron in the chrome azul S (CAS) assay, individual mutants in either *nrp* or Pbt genes exhibit no discernible defect in iron chelation (Himpsl et al., 2010). However, only the double mutant without both *nrp* and Pbt shows a noticeable inability to chelate iron even in the CAS assay. In a scenario where the colonization ability of *P. mirabilis* was tested, it was observed that neither a single mutant carrying defects in either Pbt or *nrp* nor a double mutant with impairments in both, showed any significant deficiencies in colonization when compared to the wild type (Himpsl et al., 2010). However, under conditions of co-challenge, the *P. mirabilis nrp* mutant exhibited a noteworthy impairment in colonizing the bladder and kidneys. Particularly intriguing is the observation that the *P. mirabilis nrp*/pbt double mutant displayed an impairment specifically in colonizing the kidneys, emphasizing a distinctive role for the *nrp* and proteobactin systems in renal colonization during co-challenge experiments (Himpsl et al., 2010).

### 4.2 Siderophores biosynthesis pathways

**FIGURE 7:**
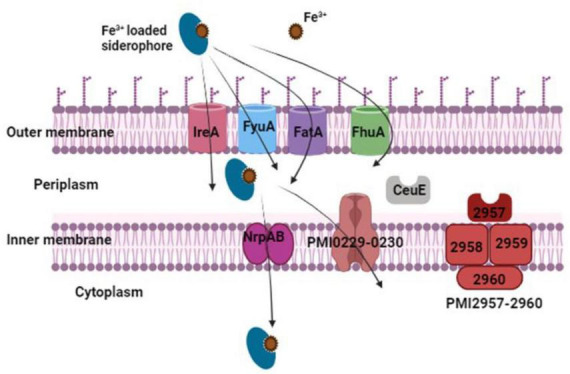
The outer membrane receptors IreA, FyuA, FatA, and FhuA transport iron-loaded siderophores. Once in the periplasm, they are transferred to the cytoplasm via the two inner membrane transport proteins PMI0229–0230 and NrpAB. The periplasmic binding protein CeuE and the inner membrane transport proteins PMI2957-2960 are two more possible iron-loaded siderophore transport proteins.

The biosynthesis of the yersiniabactin-related siderophore in *P. mirabilis* bacteria is a complex process crucial for iron acquisition. Two distinct pathways for siderophore biosynthesis are recognized in microbes, namely, the non-ribosomal peptide synthetase (NRPS) multienzyme pathway, responsible for the production of the *nrp* operon cluster, and the non-ribosomal peptide synthetase-independent (NIS) system, responsible for the production of proteobactin (Himpsl et al., 2010). Starting with the NRPS pathway, the synthesis of the yersiniabactin-related siderophore is governed by the PMI2596-2605 gene cluster. This cluster not only orchestrates the synthesis of the siderophore but also regulates its uptake and transport via ABC transporters (Figure 7). Intriguingly, this gene cluster finds its origin within the high-pathogenicity island, a genetic locus initially identified in *Yersinia pestis*. Cross-feeding experiments and biochemical analyses have demonstrated the functional distinctiveness of the yersiniabactin-related locus in *P. mirabilis*. While the bacterium is unable to utilize or produce yersiniabactin, disruption of both NRPS and NIS systems results in an *in vitro* iron-chelating defect, highlighting the production and iron-chelating activity of both siderophores. During iron limitation, *P. mirabilis* activates 45 significantly up-regulated genes associated with 21 putative iron acquisition systems. These include the TonB-ExbB-ExbD energy transducing complex, genes for heme uptake, an aerobic ferrous iron uptake system (sitABCD), two putative ferri-siderophore systems (nrpSUTABG and PMI0229-0239), and three putative ferri-siderophore transporters (ireA, PMI0331, and PMI2957-2960). Notably, the feoAB ferrous iron uptake system is not significantly upregulated during aerobic iron limitation. Conversely, genes repressed during iron limitation include predicted iron storage proteins and iron-metalloproteins. To confirm the upregulation of putative siderophore biosynthesis and ABC transport gene clusters, qPCR was employed. The *nrp* (PMI2596-2605) and *pbt* (PMI0229-0239) gene clusters, encoding yersiniabactin-related siderophore synthesis, exhibited significant up-regulation. Both clusters contain consensus sequences for Fur binding, and consistent with this, the transcription of *pbt* genes is repressed by the addition of 25 μM FeCl3⋅6H_2_O to iron chelated Lysogeny Broth (LB) medium. RT-PCR confirmed the operonic organization of both systems, with the *nrp* system consisting of two transcripts and the *pbt* system transcribed in three units (Himpsl et al., 2010).

Moving to the NIS system biosynthesis, this pathway is predicted to generate a hydroxy carboxylate siderophore. Comprising the genes PMI0229-0230, this operon expresses a two-gene transcript, encoding an ABC transport permease protein and an ABC transport ATP-binding subunit. Within this operon, *PbtI* (PMI0231) encodes a putative citrate lyase β subunit, transcribed as a single mRNA from the opposite coding strand (Himpsl et al., 2010). The enzymatic activity of citrate lyase β converts citric acid to oxaloacetate, a key biosynthetic precursor. This oxaloacetate is likely the preferred substrate for the putative siderophore biosynthesis protein encoded by *pbtA* (PMI0232) (Himpsl et al., 2010). Given the metabolic relationship of oxaloacetate to α-ketoglutarate, a recognized substrate for NIS type B synthetases, it is probable that *pbtA* also belongs to the type B subfamily of NIS synthetases (Challis, 2005). The polycistron housing the putative NIS synthetase also includes a putative TonB-dependent siderophore receptor, a putative lysine/ornithine decarboxylase, a putative pyridoxal-phosphate dependent enzyme, a putative octopine/opine/tauropine dehydrogenase, an MFS-family transporter, a substrate-binding protein, and a hypothetical protein (Himpsl et al., 2010). Indeed, all identified NIS biosynthetic pathways necessitate at least one siderophore synthetase with homology to hydroxamate synthesis enzymes IucA (NIS type A) and IucC (NIS type C) (Challis, 2005). In agreement with this, the siderophore synthetase from the aforementioned operon in *P. mirabilis* exhibits 21 and 25% identity to IucA and IucC of uropathogenic *Escherichia coli*, respectively. Further examination reveals its closest homolog in an uncharacterized siderophore system of phytopathogens *Pectobacterium* (formerly Erwinia) *carotovora subsp. atroseptica* and *P. carotovora subsp. carotovora* (54% identity to both). This *P. mirabilis* hydroxamate synthetase, along with the primary siderophore biosynthetic enzyme used by these systems, shares only 27–28% identity with the achromobactin biosynthetic enzyme AcsD, recently classified as the prototype member of a new family of enzymes, NIS type B, synthesizing a hydroxycarboxylate NIS in *Phytophthora chrysanthemi* (Münzinger et al., 2000; Franza et al., 2005; Schmelz et al., 2009). To classify the NIS synthetase subfamily represented by the synthetase of proteobactin, a phylogenetic analysis was conducted using Clustal W and 92 protein sequences homologous to siderophore synthetase PbtA, identified by BLASTp (Thompson et al., 1994). The resulting phylogeny clearly identified three known NIS subfamilies as distinct clades, placing PbtA within the same clade as Y4xN, a siderophore synthetase of *Sinorhizobium fredii* described as a type B NIS synthetase (Himpsl et al., 2010). This phylogenetic analysis suggests that the NIS of *P. mirabilis* is a type B NIS synthetase, and the genes responsible for its synthesis are denoted as pbtABCDEFGHI, representing the proteobactin synthesis (Himpsl et al., 2010).

### 4.3 Genetic characteristic of nrp in *P. mirabilis*

The *nrp* genes (PMI2597-2605) in *P. mirabilis HI4320* are situated on a pathogenicity island, as previously demonstrated (Himpsl et al., 2010). These genes exhibit homology to those involved in the synthesis of yersiniabactin, an NRPS siderophore encoded on the high-pathogenicity island (HPI) of *Yersinia* spp (Carniel et al., 1996). Within the *P. mirabilis* genomic context, the putative yersiniabactin receptor, PMI2596, is annotated as a TonB-dependent receptor, sharing 27% amino acid sequence identity with the yersiniabactin/pesticin receptor gene *psn*. The genes *PMI2597-2605* are divergently transcribed from PMI2596, constituting an operon that encodes a diverse set of proteins. This includes a major facilitator superfamily (MFS) transporter, a conserved hypothetical protein, a putative non-ribosomal peptide synthase (nrpS), two putative siderophore biosynthesis genes (*nrpU* and *nrpT*), and two putative siderophore ABC transport ATP-binding/permeases (*nrpA* and *nrpB*). Additionally, a putative 4′-phosphopantetheinyl transferase, *nrpG*, is part of this operon (Himpsl et al., 2010). To maintain nomenclature consistency, the three previously uncharacterized genes expressed in this operon are designated as *nrpX* (PMI2597), *nrpY* (PMI2598), and *nrpR* (PMI2599).

The *nrp* genes (PMI2597-2605) in *P. mirabilis HI4320*, located on a pathogenicity island as previously established, exhibit homology to yersiniabactin biosynthesis and transport genes in other Enterobacteriaceae (Himpsl et al., 2010). Despite this homology, the yersiniabactin-related siderophore synthesized by *P. mirabilis* HI4320 is predicted to lack modification by salicylate (Himpsl et al., 2010). Notably, essential genes involved in salicylic acid biosynthesis and incorporation (*pchB*, *pchA*, and *irp5* in *Pseudomonas syringae* pv. tomato DC3000 and *ybtE* and *ybtS* in *Yersinia pestis KIM*) are absent in the *P. mirabilis nrp* locus. Furthermore, the *P. mirabilis nrp* operon lacks a linked transcriptional regulator analogous to PSPTO2606 and YbtA. A distinctive feature of the *P. mirabilis nrp* operon is the presence of a conserved hypothetical protein encoded by *nrpY* (PMI2598), bearing similarity to methyltransferases found in NRPS systems. Additionally, the 4′-phosphopantetheinyl-transferase located at the end of the *P. mirabili*s *nrp* operon, *nrpG*, is encoded separately from the yersiniabactin operon on the *Y. pestis* chromosome (*ybtD*). Similarly, the 4′-phosphopantetheinyl-transferase of *P. syringae* is absent from the yersiniabactin operon (Himpsl et al., 2010).

### 4.4 The nrp-fe complex and pbtA-fe complex

The iron acquisition systems in *P. mirabilis* bacteria involve two main mechanisms. Firstly, the direct utilization of host iron compounds, including heme, hemoglobin, transferrin, and lactoferrin (Crosa, 1989). Secondly, the synthesis of siderophores and their secretion into the iron-limiting environment to scavenge extracellular iron (Crosa, 1989). Four mutants were identified with iron acquisition gene homologs, including *nrpG*, *hasR*, and genes encoding an iron transport permease and an iron-regulated outer membrane protein. The *nrp* operon, regulated by iron, contains a putative Fur box overlapping the promoter region, indicating its role in iron acquisition (Gaisser and Hughes, 1997). The high homology of nrp genes to the yersiniabactin system and the iron regulation of the nrp operon suggest the production of an undetected siderophore by *P. mirabilis* (Evanylo et al., 1984). Attempts to confirm the involvement of an α-keto acid produced by a deaminase activity in iron chelation were inconclusive. The cloned *aad* (amino acid deaminase) gene did not restore iron-limiting survival in a siderophore-negative *E. coli* strain, and CAS agar tests failed to identify iron-chelating activity in *P. mirabilis* (Massad et al., 1995). Regarding the iron acquisition proteins, hypothetical functions are based on up-regulation under iron limitation, homology with other bacterial species, and functional studies. The outer membrane receptors depend on the TonB-ExbB-ExbD complex for intracellular iron transport (Himpsl et al., 2010). Various systems, such as heme uptake, periplasmic ferrous iron import, and ferric citrate transport, are involved in iron acquisition.

During iron limitation, genes homologous to the transport and metabolism of ferri-siderophores are up-regulated in *P. mirabilis*, including putative ABC substrate-binding protein PMI0331, ABC transport system PMI2957-2960, putative iron utilization protein PMI1437, and putative esterase PMI2503. Additionally, four putative TonB-dependent ferri-siderophore receptors (ireA, PMI0363, PMI2596, and PMI0233) are up-regulated, with PMI0233 and PMI2596 located in regions encoding siderophore biosynthesis and ABC transport (Himpsl et al., 2010).

## 5 The pathogenicity due to siderophore systems and the onset of ascending UTI

The pathogenicity of siderophore systems in *P. mirabilis* during ascending UTI was investigated through transurethral inoculation experiments in murine models (Himpsl et al., 2010; Schaffer and Pearson, 2015). Siderophore synthetase and TonB-dependent receptor mutants, focusing on the proteobactin and yersiniabactin-related siderophores, were individually and co-challenged with wild-type HI4320 in the bladders of CBA/J mice. In independent infections, the loss of the proteobactin siderophore system alone or a double mutant in both siderophore synthetase genes did not attenuate the virulence of HI4320. However, the yersiniabactin-related synthetase/receptor mutant (NrpSR) demonstrated enhanced colonization compared to wild-type HI4320, with a median CFU/g slightly higher for NrpSR than HI4320. In co-challenge competition experiments, the strain lacking only proteobactin did not exhibit a fitness defect, and the loss of PbtSR may have even slightly increased fitness in the kidneys. Intriguingly, the yersiniabactin-related synthetase/receptor mutant (NrpSR) was significantly outcompeted by the wild-type in both the bladder and kidneys. Similarly, the yersiniabactin-related synthetase and proteobactin synthetase/receptor mutant (NrpSPbtSR) was significantly outcompeted by wild-type in the bladder. The consistent findings from both independent and co-challenge infections highlight the significant contribution of the yersiniabactin-related siderophore to *P. mirabilis* fitness *in vivo*. These results underscore the crucial role of siderophore systems in the pathogenicity of *P. mirabilis* during ascending UTIs (Himpsl et al., 2010). On the other hand, siderophores can be potential targets for new therapeutic approaches such as Trojan horse strategy. The latter relies on tricking the intended bacterial host into actively internalizing a compound of metallophore and antibiotic. The compound’s antibiotic component can begin acting on its target once it has entered the cell (Ghssein and Ezzeddine, 2022).

## 6 Conclusion

After an in-depth analysis of the relationship between *P. mirabilis*, pathogenicity, virulence, metal biology, and siderophores, it becomes clear that understanding bacterial infections is a complex task. A comprehensive understanding of *P. mirabilis’* pathogenic mechanisms and virulence factors is an essential step for studying its behavior within the host. Metals in general and iron in particular play a crucial role in the pathogenic journey of *P. mirabilis.* These microorganisms adeptly manipulate metal acquisition systems to obtain essential nutrients, giving them an advantage in the hostile host environment. Additionally, the importance of siderophores in metal sequestration and transport shows the adaptive nature of *P. mirabilis*. The synthesis and utilization of these small molecules not only contribute to bacterial survival but also offer potential targets for therapeutic interventions aimed at disrupting essential nutrient acquisition.

*Proteus mirabilis* is a major cause of urinary tract infections (UTIs) due to its ability to robustly evade the immune system, survive harsh conditions, form biofilms, and produce urease, contributing to stone formation and persistent infection. This literature review lays the foundation for future research on *P. mirabilis* pathogenesis. Understanding the interplay between pathogens, metals, and siderophores opens new doors for the development of innovative strategies to combat bacterial infections and lessen UTI incidents. In this ever-changing landscape of infectious diseases, the insights gained from this exploration contribute to a deeper understanding of the complex mechanisms that govern the relationship between bacteria and their hosts, offering hope for the development of targeted and effective therapeutic interventions.

## Author contributions

MC: Writing – original draft. ZH: Writing – original draft. SF: Writing – original draft. AE: Writing – original draft. ZE: Writing – review and editing, Investigation. GG: Writing – review and editing, Conceptualization.
